# Transcriptomic profiling identifies differentially expressed genes and related pathways associated with wound healing and cuproptosis-related genes in Ganxi goats

**DOI:** 10.3389/fvets.2023.1149333

**Published:** 2023-05-03

**Authors:** Lucheng Zheng, Xue Yang, Qingcan Fan, Ben Liu, Wei Hu, Yan Cui

**Affiliations:** ^1^College of Veterinary Medicine, Gansu Agricultural University, Lanzhou, China; ^2^College of Life Science and Resources and Environment, Yichun University, Yichun, China

**Keywords:** Ganxi goats, skin, wound healing, cuproptosis, transcriptome

## Abstract

**Introduction:**

Wound healing is very important for the maintenance of immune barrier integrity, which has attracted wide attention in past 10 years. However, no studies on the regulation of cuproptosis in wound healing have been reported.

**Methods:**

In this study, the skin injury model was constructed in Gnxi goats, and the function, regulatory network and hub genes of the skin before and after the injury were comprehensively analyzed by transcriptomics.

**Results:**

The results showed that there were 1,438 differentially expressed genes (DEGs), genes up-regulated by 545 and genes down-regulated by 893, which were detected by comparing day 0 and day 5 posttraumatic skin. Based on GO-KEGG analysis, DEGs that were up-regulated tended to be enriched in lysosome, phagosome, and leukocyte transendothelial migration pathways, while down-regulated DEGs were significantly enriched in adrenergic signaling in cardiomyocytes and calcium signaling pathway. There were 166 overlapped genes (DE-CUGs) between DEGs and cuproptosis-related genes, with 72 up-regulated DE-CUGs and 94 down-regulated DE-CUGs. GOKEGG analysis showed that up-regulated DE-CUGs were significantly enriched in ferroptosis, leukocyte transendothelial migration and lysosome pathways, while down-regulated DE-CUGs were significantly enriched in Apelin signaling pathway and tyrosine metabolism pathways. By constructing and analyzing of protein–protein interaction (PPI) networks of DEGs and DE-CUGs, 10 hub DEGs (ENSCHIG00000020079, PLK1, AURKA, ASPM, CENPE, KIF20A, CCNB2, KIF2C, PRC1 and KIF4A) and 10 hub DE-CUGs (MMP2, TIMP1, MMP9, MMP14, TIMP3, MMP1, EDN1, GCAT, SARDH, and DCT) were obtained, respectively.

**Discussion:**

This study revealed the hub genes and important wound healing pathways in Ganxi goats, and identified the correlation between wound healing and cuproptosis for the first time, and found that MMP2, TIMP1, MMP9, and EDN1 were the core genes associated. This study enriched the transcriptome data of wound healing in Ganxi goats and expanded the research direction of cuproptosis.

## Introduction

Ganxi goats are an excellent local breed mainly raised in the western part of Jiangxi province, with the characteristics of small body size, strong adaptability, and a high reproductive rate. The existing studies mainly focus on transport stress (1), antioxidant capacity (2), intestinal immunity (3) and genome (4), but there is no study on wound healing in Ganxi goats. Therefore, in this study, transcriptomics was used for the first time to analyze the skin on 0d and 5d of wounds in Ganxi goats, and the physiological function of skin wound healing was discussed from the perspective of mRNA.

Cuproptosis is a new pattern of programmed cell death (5), whose mechanism has attracted more and more attention. In the past year, there have been numerous reports of cuproptosis, including molecular mechanisms (6), prognostic analysis for multiple cancers (7–9), copper-induced cytotoxicity (10), and rheumatoid arthritis (11). The process is characterized by a dependence on mitochondrial respiration and a direct binding of copper to acylated components of the tricarboxylic acid cycle (TCA cycle) that results in the aggregation of acylated proteins, causing proteotoxic stress and ultimately cell death (12). Mitochondria, as an important site for aerobic respiration of cells, its damage will trigger a destructive inflammatory response in the early stages of wound healing, and promote capillary formation and pluripotent stem cell differentiation in the late stage of wound healing (13). Based on the above studies, we predict that cuproptosis may regulate wound healing through mitochondria. However, there are no studies on cuproptosis and wound healing, and the mechanism of cuproptosis in the process of wound healing is still unclear. Furthermore, more cuproptosis-related genes need to be discovered, so further research is needed.

As of now, there are many transcriptomic studies on wool (14), muscle (15) and liver (16) in other sheep breeds, including sheep, cashmere goat and small-tailed Han sheep. However, no transcriptomic studies have been conducted on Ganxi goats, and no bioinformatics studies have been conducted on the mechanism of cuproptosis-related genes in wound healing. This study explored the hub genes and key pathways associated with wound healing and cuproptosis from the perspective of mRNA through transcriptomics. Transcriptome sequencing was used to analyze DEGs of skin tissues on day 0 and day 5 after injured, and the correlation between DEGs and cuproptosis genes was analyzed to obtain DE-CUGs. Through GO-KEGG enrichment analysis of DEGs and DE-CUGs, PPI network was constructed, and genes related to hub wound healing and hub cuproptosis were identified. This study will provide new insights into cuproptosis-related genes and lead to new research possibilities. It provides sufficient omics data to analyze mRNA levels in Ganxi goats, and provides basic data and a new perspective on wound healing.

## Materials and methods

### Samples and data collection

The three Ganxi female goats used in this study were all 12 months old with a similar weight of 17.98 ± 1.15 Kg and were purchased from Yichun City, Jiangxi Province, China. The animal study was reviewed and approved by the Animal Ethics Committee of Yichun University. After anesthesia, a 10 cm surgical incision was made on the left ventral side of each Ganxi goat using sterile surgical instruments, and about 2 cm^2^ of skin was taken as a control group (FS_CK, day 0, Sample No.: FS_CK1, FS_CK2, FS_CK3). The wound was sutured without any medication. The skin was taken again at the original wound site 5 days later as an experimental group (FS_W, day 5, Sample No.: FS_W5_1, FS_W5_2, FS_W5_3), at which time the wound showed obvious granulation tissue and was initially bonded. Skin samples were stored in RNA Keeper Tissue Stabilizer (Vazyme). The transcriptome sequencing analysis of the two groups of skin was conducted, and the raw data was stored in the SRA database.^1^ The analysis of eukaryotic and transcriptome data was performed using Ensembl.^2^ A total of 2029 cuproptosis-related genes (Supplementary Table S1) were retrieved from the public GeneCards database.^3^

### Identification of DEGs and DE-CUGs

DEGs were analyzed using DESeq (version 1.30.0) and screened under the conditions of | log_2_FoldChange | > 1, significant *p*-value <0.05. DEGs between the two combinations were analyzed using the R language Pheatmap (version 1.0.8) software package. The volcano plot and heatmap showed genes distribution, genes expression multiple differences, significant result and reliable reproducibility of the data. DEGs were divided into up-DEGs and down-DEGs, and the correlation between DEGs and cuproptosis-related genes was analyzed using the website.^4^ DE-CUGs were obtained for subsequent analysis.

### Go and KEGG enrichment analysis for DEGs and DE-CUGs

The above DEGs and DE-CUGs were extracted, and the top GO was used for GO enrichment analysis. ClusterProfiler (version 3.4.4) was used for KEGG pathway enrichment analysis to identify the primary biological functions of differentially operated genes and significant enrichment pathway (the criterion for significant enrichment was *p* < 0.05, and the top 20 enrichment results are presented).

### PPI network analysis for DEGs and DE-CUGs

The STRING database^5^ was used for PPI analysis to reveal the relationship between target genes. PPI network were established based on the analysis of DEGs and DE-CUGs with a confidence score > 0.90. In the regulatory network, known proteins were labeled with protein names, and unknown proteins were labeled with Gene IDs.

### Identification of hub genes for DEGs and DE-CUGs

The CytoHubba plug-in of Cytoscape 3.9.1 was used to explore the hub genes in the regulatory network and select the top 10 hub genes.

## Result

### Identification of DEGs and DE-CUGs

**Figure 1:**
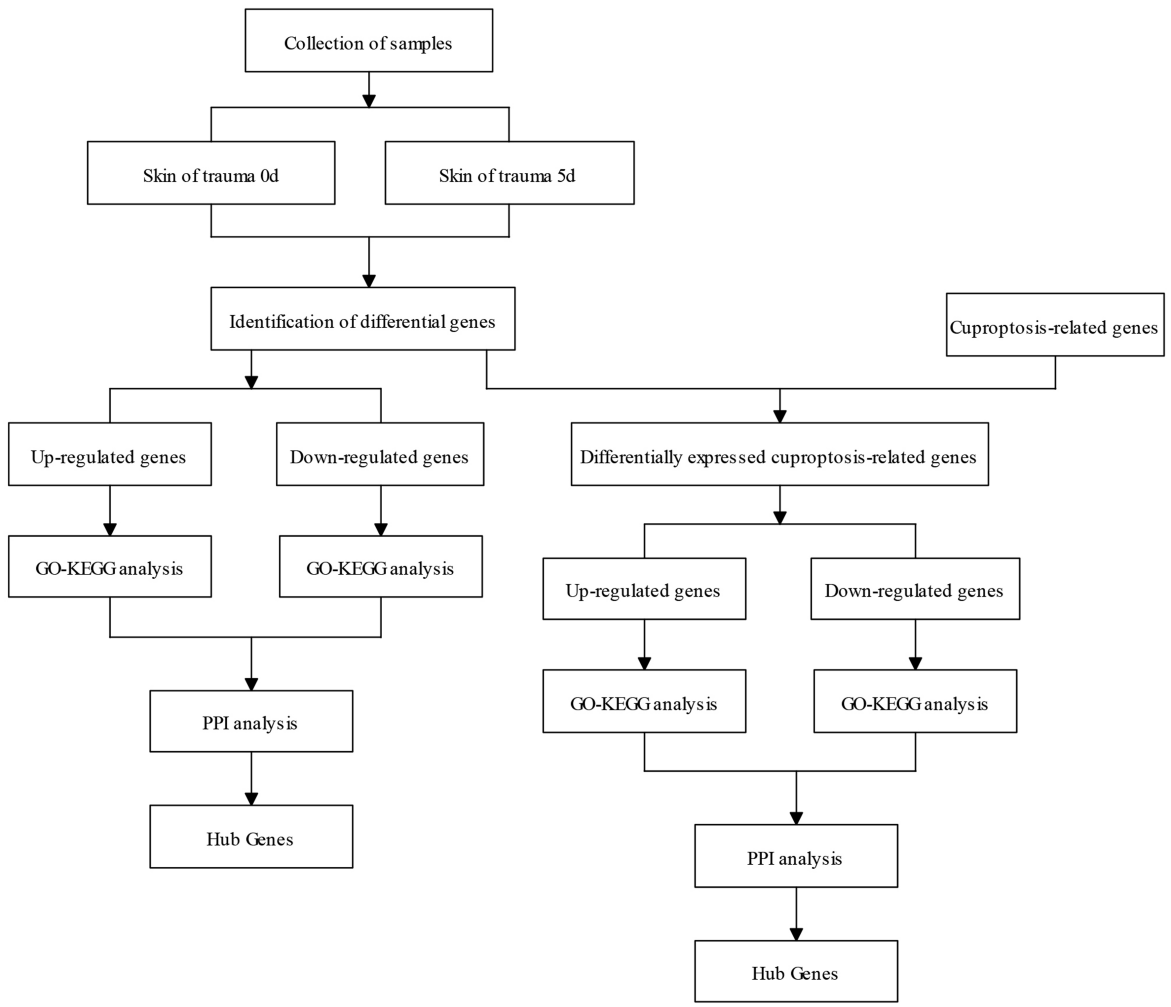
Flow chart of the research process of this study.

The flow chart of this study is shown in Figure 1.

#### Identification of DEGs

**Figure 2:**
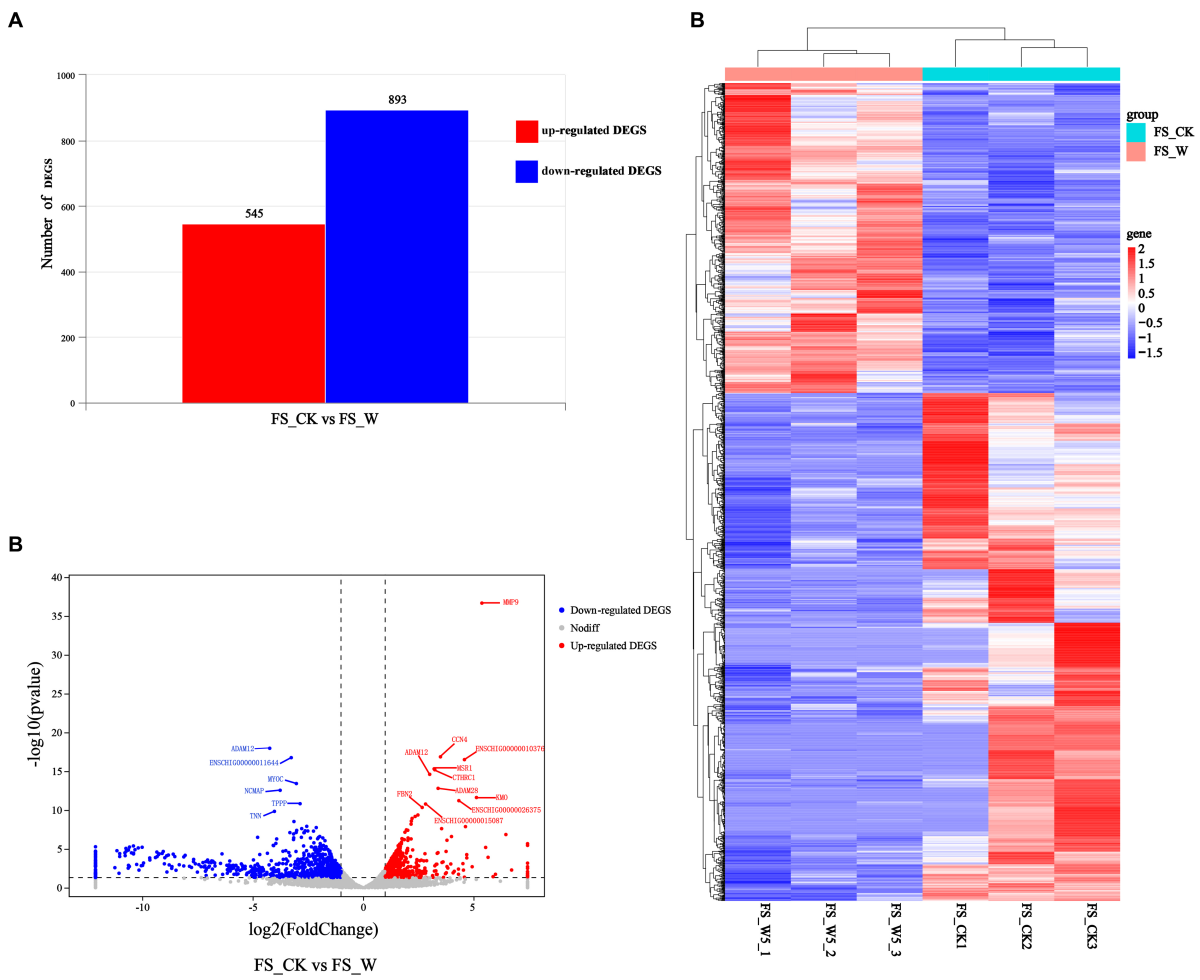
Differential expression analysis of DEGs. **(A)** Represents the numbers of up-regulated (in red) and down-regulate DEGs (in blue). **(B)** Volcano Plot visualizing the DEGs. The x axis shows log2 Fold Change, and the y axis shows-log10 (*p* value). The two vertical dashed lines in the figure represent the threshold of the expression difference fold, and the horizontal dashed lines represent the significance level threshold. Red, blue, and gray indicate genes that are up-regulated, down-regulated, or not significantly differentially expressed, respectively. **(C)** Heatmap visualizing the DEGs. Horizontal represents genes, where each column represents one sample. Red represents high expression genes, and blue represents low expression genes.

A comparison of transcriptomic data from post-injury skin on day 0 and day 5 revealed genes up-regulated by 545 and genes down-regulated by 893 (Figure 2A). The volcano plot (Figure 2B) and heatmap (Figure 2C) were also constructed. Control group (FS_CK, day 0, sample No.: FS_CK1, FS_CK2, FS_CK3) and experimental group (FS_W, day 5, sample No.: FS_W5_1, FS_W5_2, FS_W5_3) were basically symmetrical in the volcano map, and the colors were basically the same within the group, but the colors were significantly different between the groups in the heat map. These results indicated that the transcriptome data in this study had good repeatability and biological significance. Among the significantly up-regulated genes, MMP9, CCN4, MSR1, and CTHRC1 were the most prominent, while CAMK2B and MYOC were the most significantly down-regulated genes. MMP9 had an unusually high significance level and was a significantly different up-regulated gene. In addition, some significant genes were unexpectedly found to be expressed only in the skin on day 0 after injury: HJV, TRIM72, MYLK4, TMEM233, VGLL2, UCP3, PLIN5, ASB4, ADPRHL1, YIPF7, MYADML2, CHRNA1, ART1, HHATL, DUPD1, ITGB1BP2, SBK3, PLAAT1, CACNG6, ASB18, LRRC14B, MYOG, ERC2, CHRNG, P2RX6, HBAI, IQCA1L, MYOD1, ARHGAP36, PHF24, TACR3, BOLL, LBX1, SBK2, MYH8, CYTL1, MYMK, CSRNP3, HTR3A, LRRC38, SALL4. Similarly, some significant genes were found to be expressed only in skin day 5 after injury: CHRM4, MARCHF11, S100A6, TBX20, NCAN, STAB2, SYNPR, CALHM1.

#### Identification of DE-CUGs

**Figure 3:**
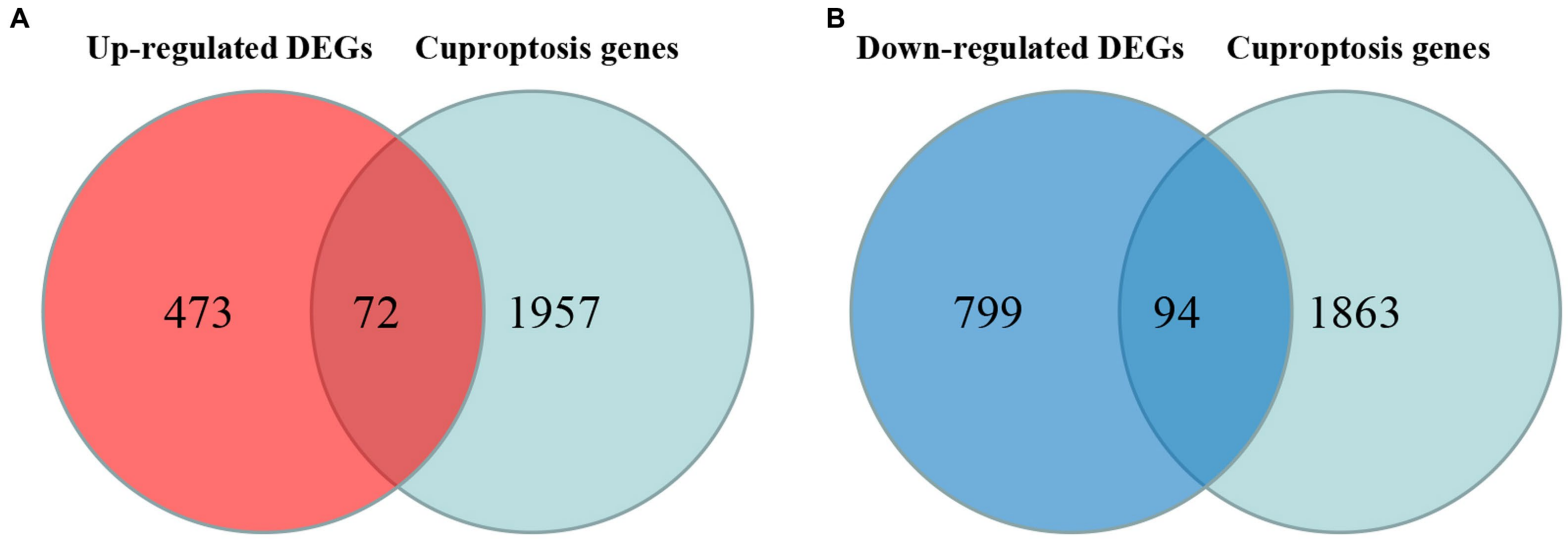
Venn diagrams for DE-CUGs. **(A)** Venn diagrams for up-regulated DE-CUGs. **(B)** Venn diagrams for down-regulated DE-CUGs. The sum of the numbers in each circle represents the total number of differentially expressed genes in each comparison group, and the overlap between the circles represents the common differentially expressed genes between the two groups.

Cross-analysis of up-regulated DEGs and down-regulated DEGs with cuproptosis-related genes was conducted, respectively. Among the DEGs, 72 up-regulated cuproptosis-related genes (Figure 3A) and 94 down-regulated cuproptosis-related genes (Figure 3B) were found. All DE-CUGs are shown in Table 1.

**Table 1:** DE-CUGs.

The 72 up-regulated DE-CUGs	The 94 down-regulated DE-CUGs
MMP9, MSR1, LOXL2, PPBP, TGFBI, ITGAM, CYBB, BMP1, NT5E, CD68, KCNN4, CTSK, TIE1, CTSB, TFPI, CTSL, CDH11, HMOX1, TIMP1, PLOD1, IGFBP4, PRND, FTH1, MMP14, ITGB2, FN1, SLC46A1, TYMS, GPR18, P4HB, PCDH12, MDK, SLC31A2, LTBP1, SLC2A10, SPARC, COL4A1, MMP2, LGALS1, SCN3A, JAK3, CHI3L1, NQO2, AKR1B1, KYNU, TLR7, TF, KRT3, TRPM2, NEU1, MMP1, TG, S100A6, PLAU, GAL, CLEC6A, BRCA2, SCAMP5, CDC45, CYP27B1, CLEC4E, CDC6, FAH, AHSG, HGF, CDKN3, CTSH, CYBA, ADA, TNFRSF1B, CP, CTLA4	PON3, TPPP3, LTBP4, TIMP3, KRT82, GPC3, HJV, MYL2, CCL26, SLAIN1, KRT8, CA3, ARSH, MB, ACTC1, SCN4B, CA4, FMO3, S100A1, GSN, SLC6A4, RYR1, NOS1, GRIA3, SCD, MSX1, ALDH1A1, DCT, AQP5, FASN, KCNJ11, TRIM63, CYP2F1, COX6A2, ACHE, CACNA1S, NCAM1, SARDH, COL8A2, PPARGC1A, CCL27, GLI1, NTRK2, GJB1, CAV3, TYRP1, OCA2, CCND1, SCN4A, GCAT, COL2A1, SNCA, TIMP4, MT4, ACE, PER1, TYR, MGP, PROM1, AGTR1, AOC1, AOX1, CRYAB, TERT, FLNC, ACTN3, SLC4A4, CUX2, CASQ2, PDP1, RXRG, NALCN, FASLG, ZBTB16, NDP, MOXD1, MAT1A, COQ8A, PLA2G2E, NEXN, SLC2A4, BCHE, LRP2, CCK, ESR1, HTR3A, PRKAA2, DUSP1, PTCH1, TNFSF10, SLC45A2, EDN1, TMOD1, SLC6A1

### Enrichment of DEGs and DE-CUGs

**Figure 4:**
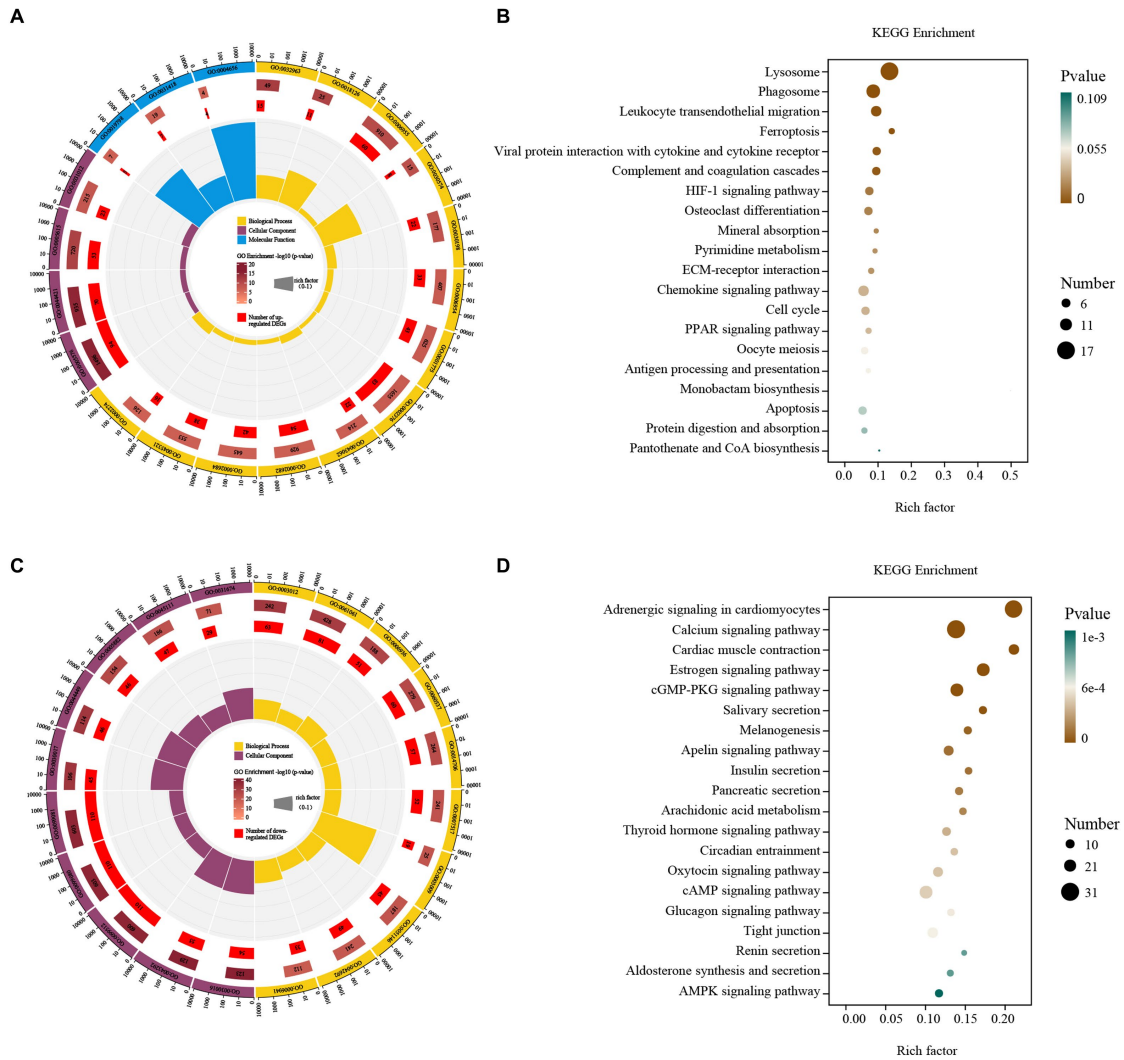
GO and KEGG enrichment analysis of the DEGs. GO enrichment analysis of up- **(A)** and down-regulated **(C)** DEGs. The outermost circle represents the classification of the GO Term, indicated by color, and the GOID is represented by the text; The middle circle represents the level of GO Enrichment, indicated by-log10 (*p*-value), and the number in the circle represents the total number of genes annotated to that GO Term; The inner circle number represents the number of up-or down-regulated DEGs; The high and low levels of the fan bar chart indicate the rich factor, which is the ratio of the number of up-or down-regulated DEGs to the total number of genes annotated to the GO Term. KEGG enrichment analysis of up- **(B)** and down-regulated **(D)** DEGs. The high and low levels of the fan bar chart indicate the rich factor, which is the ratio of the number of up-or down-regulated DEGs to the total number of genes annotated to the GO Term; The size of the dot in the figure indicates the number of up-or down-regulated DEGs in the corresponding pathway, and the depth of color indicates the level of significance.

Up-regulated DEGs were evaluated by enrichment analysis after skin comparison between day 0 and day 5 afterinjury. Pathway enrichment scores derived from GO and KEGG were used to predict gene phenotypes on up-regulated DEGs. The results showed that the up-regulated components of DEGs were extracellular region, extracellular region part, extracellular space and extracellular matrix. The biological processes covered collagen metabolic process, protein hydroxylation, immune response, collagen catabolic process, extracellular matrix organization, inflammatory response, cell activation, immune system process, extracellular structure organization, regulation of immune system process, positive regulation of immune system process, leukocyte activation, myeloid leukocyte activation. The molecular function included procollagen-proline dioxygenase activity, L-ascorbic acid binding and procollagen-proline 4-dioxygenase activity (Figure 4A). These up-regulated DEGs were lysosome, phagosome, leukocyte transendothelial migration, ferroptosis, viral protein interaction with cytokine and cytokine receptor, complement and coagulation cascades, HIF-1 signaling pathway, osteoclast differentiation, mineral absorption, pyrimidine metabolism, ECM-receptor interaction, chemokine signaling pathway, cell cycle, PPAR signaling pathway, oocyte meiosis, antigen processing and presentation, monobactam biosynthesis, apoptosis, protein digestion and absorption, pantothenate and CoA biosynthesis (Figure 4B).

Down-regulated DEGs were evaluated by GO-KEGG analysis to predict their phenotype. The cellular components were myofibril, contractile fiber, supramolecular fiber, supramolecular complex, supramolecular polymer, sarcomere, contractile fiber part, intermediate filament, intermediate filament cytoskeleton and I band. The biological processes covered muscle system process, muscle structure development, muscle contraction, muscle tissue development, striated muscle tissue development, muscle organ development, skeletal muscle contraction, striated muscle cell differentiation, muscle cell differentiation and striated muscle contraction (Figure 4C). These down-regulated DEGs included adrenergic signaling in cardiomyocytes, calcium signaling pathway, cardiac muscle contraction, estrogen signaling pathway, cGMP-PKG signaling pathway, salivary secretion, melanogenesis, apelin signaling pathway, insulin secretion, pancreatic secretion, arachidonic acid metabolism, thyroid hormone signaling pathway, circadian entrainment, oxytocin signaling pathway, cAMP signaling pathway, glucagon signaling pathway, tight junction, renin secretion, aldosterone synthesis and secretion and AMPK signaling pathway (Figure 4D).

**Figure 5:**
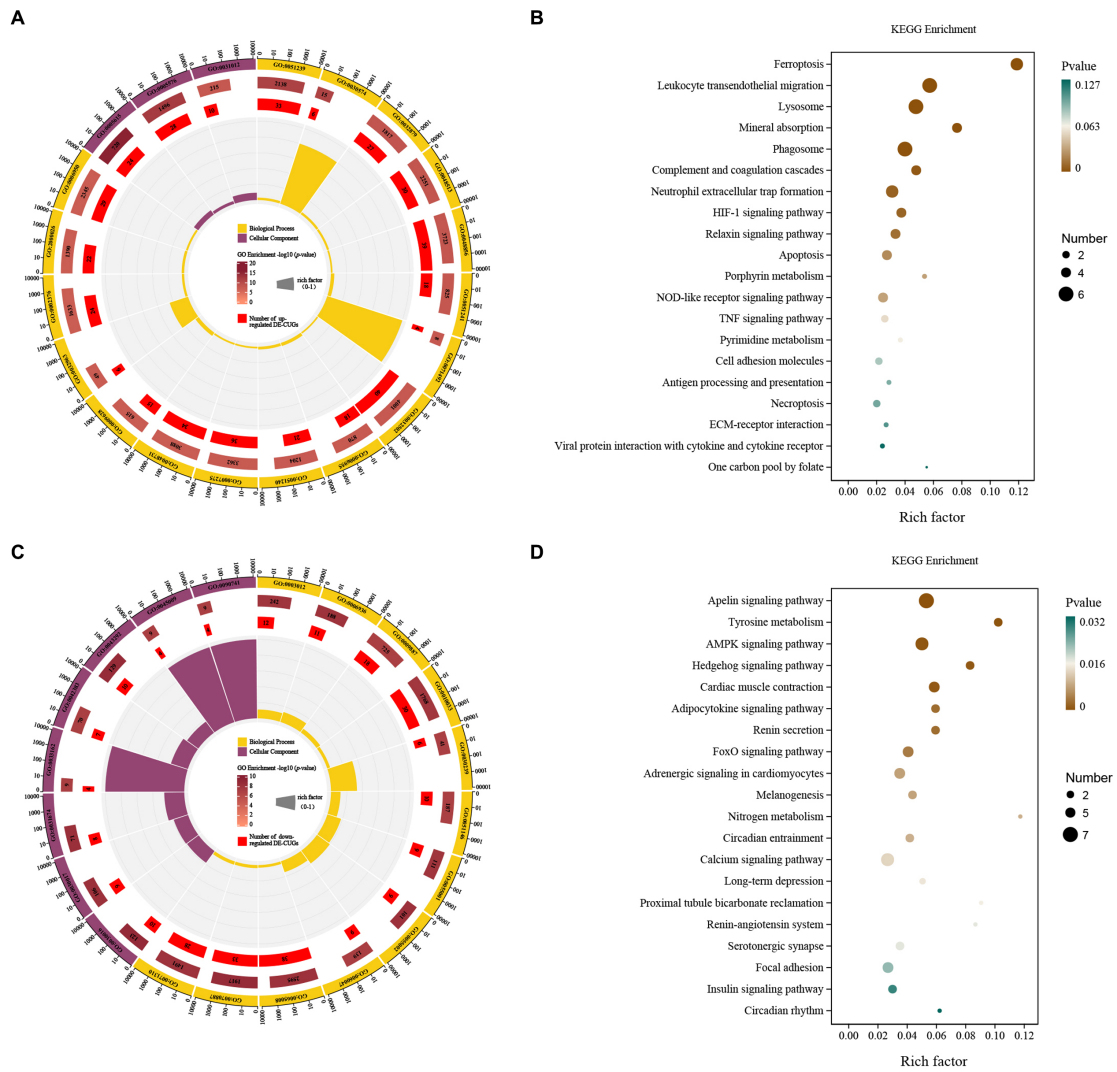
GO and KEGG pathway enrichment analysis of the DE-CUGs. GO enrichment analysis of up- **(A)** and down-regulated **(C)** DE-CUGs. The outermost circle represents the classification of the GO Term, indicated by color, and the GOID is represented by the text; The middle circle represents the level of GO Enrichment, indicated by-log10 (*p*-value), and the number in the circle represents the total number of genes annotated to that GO Term; The inner circle number represents the number of up-or down-regulated DE-CUGs; The high and low levels of the fan bar chart indicate the rich factor, which is the ratio of the number of up-or down-regulated DE-CUGs to the total number of genes annotated to the GO Term. KEGG enrichment analysis of up- **(B)** and down-regulated **(D)** DE-CUGs. The high and low levels of the fan bar chart indicate the rich factor, which is the ratio of the number of up-or down-regulated DE-CUGs to the total number of genes annotated to the GO Term; The size of the dot in the figure indicates the number of up-or down-regulated DE-CUGs in the corresponding pathway, and the depth of color indicates the level of significance.

The up-regulated cross genes (up-DE-CUGs) were obtained by comparing the correlation between up-DEGs and cuproptosis-related genes. Up-DE-CUGs were evaluated by GO-KEGG analysis to predict their phenotype. The cellular components were extracellular space, extracellular regio and extracellular matri. The biological processes covered regulation of multicellular organismal process, collagen catabolic process, regulation of localization, animal organ development, anatomical structure development, negative regulation of multicellular organismal processes, cellular response to UV-A, developmental process, immune response, positive regulation of multicellular organismal processes, multicellular organism development, system development, response to abiotic stimulus, collagen metabolic process, immune system process, regulation of multicellular, rganismal development and response to stress (Figure 5A). These up-regulated DE-CUGs included the ferroptosis, leukocyte transendothelial migration, lysosome, mineral absorption, phagosome, complement and coagulation cascades, neutrophil extracellular trap formation, HIF-1 signaling pathway, relaxin signaling pathway, apoptosis, porphyrin metabolism, NOD-like receptor signaling pathway, TNF signaling pathway, pyrimidine metabolism, cell adhesion molecules, antigen processing and presentation, necroptosis, ECM-receptor interaction, viral protein interaction with cytokine and cytokine receptor, and one carbon pool by folate (Figure 5B).

The down-regulated cross genes (down-DE-CUGs) were obtained by comparing the correlation between down-DEGs and cuproptosis-related genes. Down-DE-CUGs were evaluated by GO-KEGG analysis to predict their phenotype. The cellular components were myofibril, contractile fiber, I band, sarcomere, melanosome membrane, chitosome, pigment granule membrane and sarcolemma. The biological processes covered cellular response to chemical stimulus, striated muscle cell development, regulation of biological quality, cellular response to organic substance, muscle contraction, muscle system process, muscle cell development, response to organic substance, heart contraction, myofibril assembly, striated muscle cell differentiation and animal organ morphogenesis (Figure 5C). These down-regulated DE-CUGs included the Apelin signaling pathway, Tyrosine metabolism, AMPK signaling pathway, Hedgehog signaling pathway, Cardiac muscle contraction, adipocytokine signaling pathway, renin secretion, FoxO signaling pathway, adrenergic signaling in cardiomyocytes, melanogenesis, nitrogen metabolism, circadian entrainment, calcium signaling pathway, long-term depression, proximal tubule bicarbonate reclamation, renin-angiotensin system, serotonergic synapse, focal adhesion, insulin signaling pathway and circadian rhythm (Figure 5D).

### PPI network analysis of DEGs and DE-CUGs

**Figure 6:**
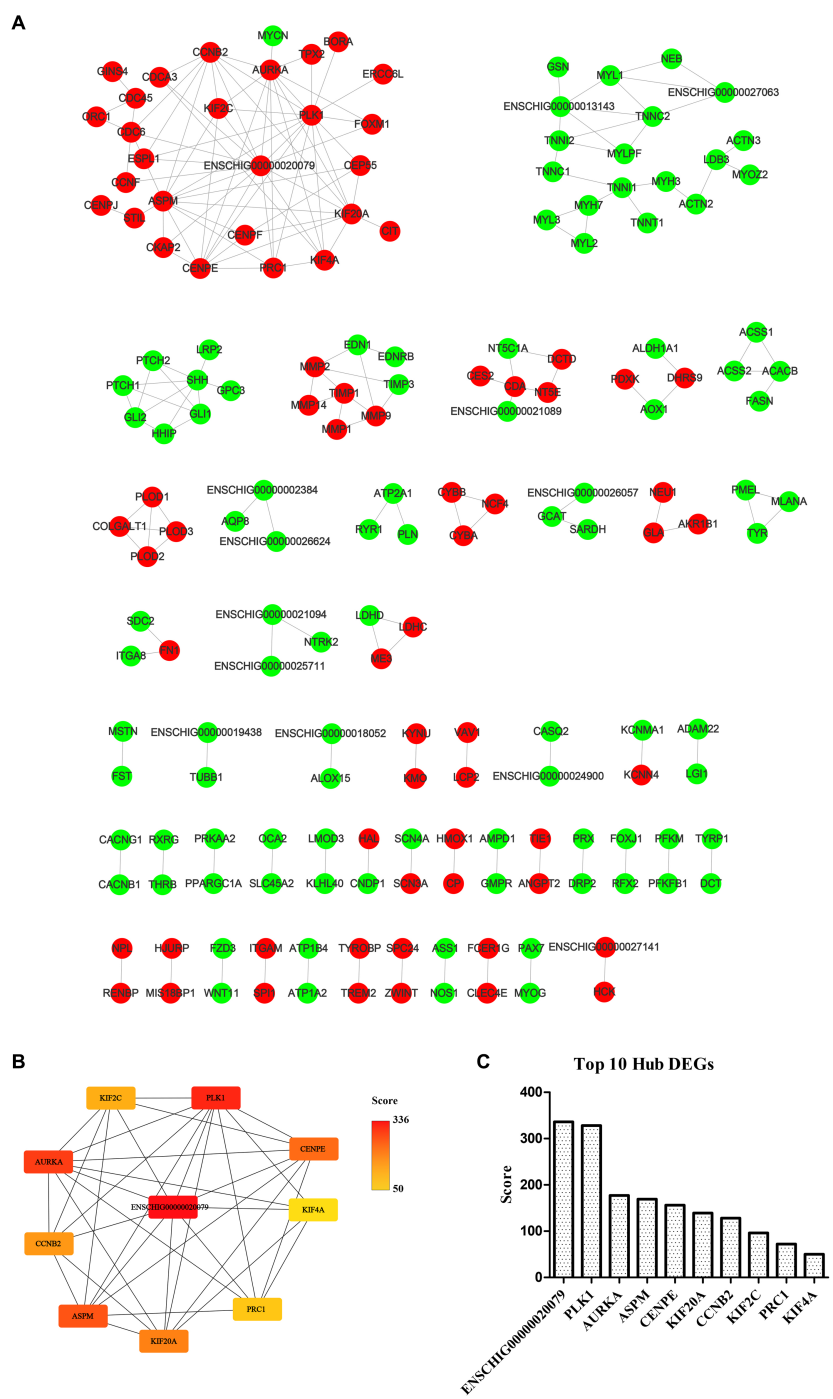
PPI network and hub analysis of DEGs. **(A)** PPI network of DEGs. Dots in green represent down-regulated genes, while dots in red represent up-regulated genes. **(B)** PPI network of hub DEGs. The depth of color indicates the level of significance. **(C)** The rank of hub DEGs.

**Figure 7:**
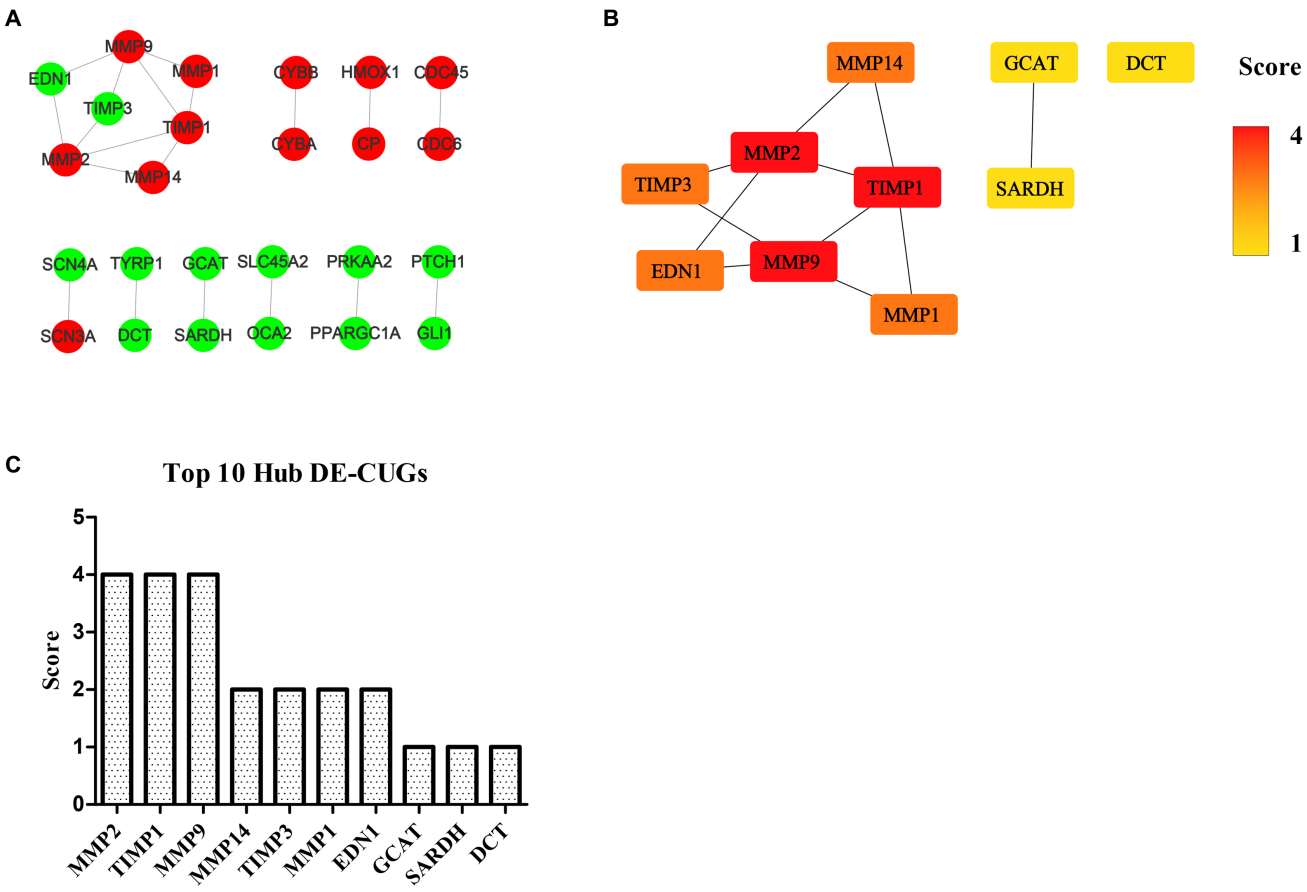
PPI network and hub analysis of DE-CUGs. **(A)** PPI network of DE-CUGs. Dots in green represent down-regulated genes, while dots in red represent up-regulated genes. **(B)** PPI network of hub DE-CUGs. The depth of color indicates the level of significance. **(C)** The rank of hub DE-CUGs.

The STRING database (See Footnote 5) was utilized for protein interaction analysis of DEGs and DE-CUGs, and two PPI networks were constructed. 194 PPI associated gene pairs (Figure 6A) and 19 (Figure 7A) were obtained, respectively. In the PPI network of DEGs, 76 up-regulated DEGs and 98 down-regulated DEGs (Table 2) were identified. The core genes were ENSCHIG00000020079 (an unknown Gene, named after Gene ID) and PLK1, each regulating 15 genes, followed by ASPM with 12 genes, AURKA with 11 genes, and KIF20A with 10 genes (Table 3). In the DE-CUGs PPI network, 12 up-regulated DEGs (CDC45, CDC6, CP, CYBA, CYBB, HMOX1, MMP1, MMP14, MMP2, MMP9, SCN3A and TIMP1) and 13 down-regulated DEGs (DCT, EDN1, GCAT, GLI1, OCA2, PPARGC1A, PRKAA2, PTCH1, SARDH, SCN4A, SLC45A2, TIMP3, and TYRP1) were identified. MMP2 regulates EDN1, TIMP3, TIMP1, and MMP14. MMP9 regulates EDN1, TIMP3, TIMP1, and MMP1. TIMP1 regulates MMP2, MMP9, MMP1, and MMP14.

**Table 2:** DEGs in PPI network.

The 76 up-regulated DEGs	The 98 down-regulated DEGs
MMP9, KMO, PLOD2, ITGAM, CYBB, KIF20A, NT5E, TREM2, CENPE, KCNN4, ENSCHIG00000027141, COLGALT1, PLOD3, TIE1, PLK1, HMOX1, HJURP, AURKA, NPL, TIMP1, PLOD1, MMP14, SPC24, FCER1G, ASPM, CCNF, FN1, KIF4A, CEP55, CDCA3, VAV1, TYROBP, ESPL1, PRC1, ENSCHIG00000020079, RENBP, GLA, CKAP2, TPX2, MMP2, KIF2C, CENPF, SCN3A, DCTD, CIT, AKR1B1, KYNU, ZWINT, MIS18BP1, NEU1, CDA, ANGPT2, CCNB2, MMP1, GINS4, CENPJ, CDC45, STIL, BORA, ORC1, DHRS9, CLEC4E, CDC6, ERCC6L, FOXM1, SPI1, LCP2, LDHC, ME3, CES2, CYBA, PDXK, CP, HCK, HAL, NCF4	WNT11, TIMP3, MYH7, GPC3, PTCH2, ACTN2, ENSCHIG00000026057, PLN, MYL2, SDC2, ASS1, TNNC1, TNNT1, PRX, TNNC2, LDB3, ITGA8, CACNG1, ATP2A1, GSN, LGI1, RYR1, FST, NOS1, ENSCHIG00000024900, LMOD3, HHIP, TUBB1, ALDH1A1, SHH, ENSCHIG00000027063, DCT, MYLPF, AMPD1, FASN, KLHL40, CNDP1, MYOZ2, ACSS2, ENSCHIG00000018052, ENSCHIG00000013143, AQP8, ACSS1, ENSCHIG00000021094, GLI2, MYCN, ENSCHIG00000002384, SARDH, PPARGC1A, MYL1, ENSCHIG00000026624, TNNI2, GLI1, NTRK2, PAX7, PFKFB1, MYOG, THRB, TYRP1, OCA2, PMEL, SCN4A, ATP1A2, GCAT, MSTN, TNNI1, MLANA, NEB, TYR, RFX2, EDNRB, ALOX15, KCNMA1, AOX1, ENSCHIG00000021089, ENSCHIG00000025711, GMPR, ACTN3, ATP1B4, DRP2, FOXJ1, CASQ2, RXRG, PFKM, LDHD, NT5C1A, ADAM22, FZD3, NSCHIG00000019438, LRP2, PRKAA2, CACNB1, PTCH1, MYH3, MYL3, ACACB, SLC45A2, EDN1

**Table 3:** The top 5 genes with the widest regulatory range in the DEGs PPI network.

Core gene	Interacting genes
PLK1	AURKA, ESPL1, CENPE, ASPM, PRC1, CEP55, ERCC6L, CCNB2, BORA, KIF4A, KIF2C, TPX2, ENSCHIG00000020079, FOXM1
ENSCHIG00000020079	CDCA3, ESPL1, CDC6, ASPM, PRC1, CCNF, FOXM1, KIF4A, AURKA, CCNB2, KIF20A, KIF2C, PLK1, CKAP2,CENPE
ASPM	PLK1, CCNB2, PRC1, CENPF, ENSCHIG00000020079, ESPL1, CKAP2, KIF20A, STIL, CEP55, KIF2C, CENPE
AURKA	PLK1, KIF20A, KIF2C, MYCN, FOXM1, CENPE, KIF4A, PRC1, TPX2, ENSCHIG00000020079, CCNB2
KIF20A	AURKA, KIF4A, CENPF, CENPE, PLK1, ASPM, CCNB2, CIT, CEP55, ENSCHIG00000020079

### Hub DEGs and DE-CUGs

The genes in PPI networks of DEGs and DE-CUGs were screened using the cytoHubba plug-in in Cytoscape 3.9.1, and the top 10 hub DEGs/DE-CUGs were obtained. The PPI network of hub DEGs (Figure 6B) and its ranking were as follows (Figure 6C): ENSCHIG00000020079, PLK1, AURKA, ASPM, CENPE, KIF20A, CCNB2, KIF2C, PRC1, and KIF4A. ENSCHIG00000020079 and PLK1 are the most important hub genes in wound healing, and their significance requires further verification. The PPI network of hub DE-CUGs (Figure 7B) and its ranking were as follows (Figure 7C): MMP2, TIMP1, MMP9, MMP14, TIMP3, MMP1, EDN1, GCAT, SARDH and DCT. MMP2, TIMP1, and MMP9 were the most important hub genes for cuproptosis to regulate wound healing, which need further verification.

## Discussion

In this study, we identified the central genes and pathways of wound healing by comparing the skin samples on day 0 and day 5 after injury. We further discussed the hub genes and pathways of cuproptosis-related genes that regulate the wound healing process in Ganxi goats. It was found that MMP9, CAMK2B, CCN4, MSR1, and CTHRC1 were the top 5 genes with a significant difference between the two groups of skin samples. The difference factor of MMP-9 was much higher than that of the other genes. MMP-9 was also identified as a key gene in wound healing in previous studies. Mmp-9 can enhance keratinocyte migration, which is essential for re-epithelialization and the completion of wound healing (17). Moreover, MMP-9 cleaves several extracellular matrix (ECM) proteins and regulates ECM remodeling (18). During inflammation, prostaglandin E2 up-regulates the expression of MMP-9, inducing dendritic cell migration and initiating immune responses (19). The concentration of active MMP-9 increases with wound severity and infection (17). Silencing MMP-9 promotes wound healing in diabetic patients (20). All of the aforementioned studies on MMP-9 in wound healing or infection indicated that the expression of MMP-9 was regulated by injury, which is consistent with the fact that MMP-9 is a key gene in wound healing of Ganxi goats. In this study, lysosomal and phagocytic pathways were found to be the key pathways for wound healing. Wound healing is a coordinated process that relies initially on proinflammatory macrophages and subsequently on the prolytic function of these cells (13). Lysosomes are important regulatory platforms for vesicular transport pathways such as endocytosis, phagocytosis, and autophagy, capable of breaking down a wide range of endogenous and exogenous substances such as macromolecules, certain pathogens, and old or damaged organelles (21). A large number of research achievements have been accumulated on lysosomes and phagocytes, which are widely involved in the protein degradation and structural remodeling process. These mechanisms are consistent with the wound healing mechanism observed in Ganxi goats. In the hub gene test, ENSCHIG00000020079 and PLK1 showed key regulatory roles, especially the unknown gene ENSCHIG00000020079, which played the most important role and has great research value. Further studies may reveal differences in wound healing mechanisms between ruminants and other animals, such as humans and mice. PLK1 is a key regulator of cell mitosis and is overexpressed in various of cancers (22). Song et al. found that interference with PLK1 by siRNA in wound healing and invasion experiments can significantly inhibit cell migration and invasion (23). In the study on the mechanism of Yunnan Baiyao in the treatment of Staphylococcus aureus infection revealed by RNA sequencing, Zhang et al. identified 10 key regulatory genes by constructing protein interaction network, among which PLK1 was also included (24). In Yang et al.’s study on biomarkers of medulloblastoma, PLK1 and CENPE were also identified as hub genes (25). The above results are consistent with the findings of this study, and the screened hub genes play an important role in multiple pathways, including wound healing.

Cuproptosis is a newly-discovered form of cupric-induced programmed cell death dependent on mitochondrial respiration, which is different from all other known programmed cell death. Cuproptosis leads to protein aggregation through copper binding to lipoacylase in the tricarboxylic acid cycle, which triggers proteotoxic stress and ultimately leads to cell death (7). The relationship between mitochondrial damage and cuproptosis has attracted extensive attention from scholars, but no high-throughput studies have been conducted to investigate the potential association between cuproptosis and wound healing, as well as the mechanism of cuproptosis in wound healing. In this study, we obtained 166 DE-CUGs, including 72 up-regulated DE-CUGs and 94 down-regulated DE-CUGs, by cross-analysis of wound healing differential genes and cuproptosis-related genes. GO-KEGG enrichment analysis of DE-CUGs showed that the up-regulated DE-CUGs were significantly enriched in ferroptosis, leukocyte transendothelial migration and lysosome signaling pathways, while down-regulated DE-CUGs were mainly involved in Apelin signaling pathways. Transient lysosomal leakage events play an important role in regulating mitochondrial function and metabolism to maintain homeostasis (26). The processes of apoptosis, inflammation and tissue repair associated with wound healing are all dependent on lysosomes. The lysosome pathway may play an indirect role in the regulation of cuproptosis on wound healing. Finally, by constructing the DE-CUGs PPI network and screening for hub genes, this study found that the MMPs/TIMPs system was the most important hub gene set, including MMP9, MMP1, MMP2, MMP14, TIMP1, and TIMP3, which were completely present in the hub analysis results of wound healing-and cuproptosis-related genes. This could be an important signaling pathway, and among them, MMP2, MMP9, and TIMP1 play a core role. Matrix metalloproteinases (MMPs) are a type of internal peptidase that depends on zinc ions to function, can degrade the basement membrane and extracellular matrix, and play an important role in physiological processes such as wound healing (27). TIMPs are specific inhibitors of MMPs. TIMP-1 is produced by macrophages and connective tissue cells, while TIMP-3 exists only in ECMs and can induce cell apoptosis (28). The imbalance of dynamic MMPs/TIMPs expression is associated with many diseases, such as liver fibrosis (29), wound healing disorders (30), and multiple cancers (31), which are often associated with cuproptosis (7). Wound excipients containing copper and zinc have been shown to promote fibroblast proliferation, increase collagen fibers, new arterial and venous capillaries, and enhance the wound healing process (32). The aforementioned study demonstrates a possible link between wound healing and cuproptosis through copper and zinc, which is consistent with our results but requires further research. Another exciting finding is that EDN1, which was screened in the hub DE-CUGs, directly interacts with MMP2 and MMP9. This can be used as direct evidence of the association between wound healing and cuproptosis. There are evidences suggesting that EDN1 can indeed mediate mitochondrial oxidative stress and dysfunction. Daehn et al. found that EDN1 mediates mitochondrial oxidative stress in glomerular endothelial cells, leading to podocyte depletion and glomerulosclerosis (33). The study highlights the important role of endothelial mitochondrial oxidative stress in the pathogenesis of focal segmental glomerular sclerosis (FSGS) and identifies EDN1 as a key mediator of this process. Haiying Qi et al. identified a relationship between glomerular endothelial mitochondrial dysfunction and increased levels of glomerular EDN1 receptor type A (Ednra) expression and circulating EDN1 (34). This discovery further supports our previous hypothesis that cuproptosis may regulate wound healing through mitochondria, providing a fresh perspective for the investigation of cuproptosis in wound healing.

In this study, skin wound healing of Ganxi goats was investigated, and an unknown gene, ENSCHIG00000020079, was discovered to play the most critical regulatory role in wound healing. This new gene provides a new direction for further reserch. Key genes, including MMP2, MMP9, TIMP1, and EDN1, that regulate wound healing via cuproptosis were identified. The lysosome pathway was found to be a crucial pathway for wound healing and cuproptosis. The exploration of these key genes and pathways provides new targets for the study of wound healing and new transcriptome data for the investigation of cuproptosis-regulated genes related to wound healing.

## Data availability statement

The datasets presented in this study can be found in online repositories. The names of the repository/repositories and accession number(s) can be found at: https://www.ncbi.nlm.nih.gov/, PRJNA922517.

## Ethics statement

The animal study was reviewed and approved by the Animal Ethics Committee of Yichun University. Written informed consent was obtained from the owners for the participation of their animals in this study.

## Author contributions

LZ and YC conceived and planned the experiment. LZ carried out the experiment. XY processed the data. QF, BL, and WH participated in experiment. All authors contributed to the article and approved the submitted version.

## Funding

This research was supported by the National Natural Science Foundation of China (Grant No. 31972634) and the Science and Technology Research Project of Education Department of Jiangxi Province (Grant No. GJJ170918).

## Conflict of interest

The authors declare that the research was conducted in the absence of any commercial or financial relationships that could be construed as a potential conflict of interest.

## Publisher’s note

All claims expressed in this article are solely those of the authors and do not necessarily represent those of their affiliated organizations, or those of the publisher, the editors and the reviewers. Any product that may be evaluated in this article, or claim that may be made by its manufacturer, is not guaranteed or endorsed by the publisher.

## Footnotes

^1^ https://www.ncbi.nlm.nih.gov/sra/PRJNA922517

^2^ https://asia.ensembl.org/Capra_hircus

^3^ https://www.genecards.org/

^4^ http://bioinformatics.psb.ugent.be/webtools/Venn/

^5^ https://string-db.org/
