# Supplementary material for: Transcriptomic profiling identifies differentially expressed genes and related pathways associated with wound healing and cuproptosis-related genes in Ganxi goats
Source: Front Vet Sci. 2023 May 3;10:1149333. doi: 10.3389/fvets.2023.1149333 (PMC10259478; doi:10.3389/fvets.2023.1149333)
Supplement: Supplementary file 1 [file Table_1.docx]

Supplementary Material

Bioinformatic profiling identifies potential cuproptosis-related genes of wound healing in Ganxi goats skin

Lucheng Zheng^1,2^, Xue Yang^2^, Qingcan Fan^2^, Ben Liu^2^, Wei Hu^2^, Yan Cui^1*^

*** Correspondence:** yancui@gsau.edu.com

# Supplementary Table 1

SUPPLEMENTARY TABLE 1 2029 cuproptosis-related genes

| TP53, MET, CASP8, TNF, FAS, ATP7B, BCL2, EGFR, SCN5A, TP7A, AKT1, PTEN, CASP3, ATM, IL6, HIF1A, PIK3CA, FASLG, ADA, SOD1, JAK3, MYC, BAX, CDKN2A, KRAS, IFNG, STAT3, CTNNB1, CCND1, MAPK1, BRAF, VEGFA, XIAP, MTOR, RAG1, JUN, TGFB1, ERBB2, CDH1, HRAS, APP, PTCH1, CXCL8, MAPK8, TNFSF10, IL1B, VHL, IL10, NOS2, MIR21, RAG2, CDKN1A, CP, BRCA1, TERT, BCL2L1, RIPK1, CD4, ICAM1, HBB, CD274, IL2, BRCA2, NFKB1, LMNA, PARP1, STAT1, KITLG, AIFM1, BAD, INS, PTPRC, SLC2A1, MAP2K1, SETD2, MAPK14, ABL1, PPARG, RYR2, CXCR4, CYCS, CTLA4, JAK2, SRC, ALK, IL4, ALB, VCAM1, ESR1, PIK3CG, CASP9, RAF1, PRNP, EGF, CAV3, PTGS2, NFKBIA, MCL1, HLA-DRB1, PIK3R1, PCNA, CDKN1B, CAT, FGF2, IL2RA, NFE2L2, ITGB1, CCL2, ATOX1, TNFRSF1A, CD44, CD40, BIRC5, KDR, KCNQ1, FLT3, IL1A, PTPN11, PECAM1, HNF1A, PRKN, MMP9, TLR4, MDM2, RYR1, RAC1, SLC31A1, IL3, SP1, FOS, IGF1, AR, HMOX1, CD40LG, SCO2, SNCA, IL13, CACNA1C, MAPT, RELA, CXCL12, NCAM1, CCS, ABCB1, MAPK3, TFRC, IRF1, IKBKB, AOC3, SYK, MMP2, CD28, NOS3, MSH2, ITGB3, CDK1, CCL5, COMMD1, TYR, VIM, CALM1, HLA-A, APOE, HGF, FGFR1, CAV1, CSF3, APC, CD36, BCL2L11, TLR2, EPO, HSP90AA1, MUC1, DAXX, IKBKG, COX17, MITF, IL7, CSF2, C11orf65, GJA1, MIR200C, ANXA5, BIRC3, KCNH2, LOX, NGF, WT1, IFNA1, CREB1, BDNF, TP73, EIF2AK3, SMAD4, IL18, CDC42, CDKN3, FOXO1, CLU, SOD2, CASP1, FH, FN1, ITGB2, CASP7, HMGB1, F2, G6PD, CALR, SPP1, BTK, DDIT3, RUNX1, MLH1, MAPK10, GFAP, NLRP3, NTRK1, HSPA5, OGG1, CSF1, EGR1, SELE, CCR5, SERPINE1, CPT2, MMP1, GAPDH, THPO, BMP2, SQSTM1, TRDN, CASQ2, LEP, IL5, CXCL10, CRP, B2M, PSEN1, INSR, EPAS1, PLAU, PDGFB, ITGA4, PARK7, BAK1, PRKD1, DNMT1, CDH2, E2F1, DBH, LIF, CYP1A1, MPO, CCNA2, IGF2, CCL3, KCNJ11, BIRC2, CFTR, CHUK, GSK3B, CCNB1, VWF, ELN, SMPD1, MIR205, NFKB2, SELP, SOX9, FOXO3, EZH2, EDN1, ACE, CEACAM5, BMP6, HDAC9, NGFR, F3, SDHB, FBN1, CD79A, KCNE1, AOC1, MME, HSPA4, ZEB1, SST, ERBB4, NPC1, MIR200B, TNFRSF1B, SLC16A1, KRT18, GSTP1, CCR6, TF, MIR223, TH, ANK2, AFP, NQO1, CXCR3, HFE, ITGAM, ENO2, NUP214, HSPB1, IGFBP3, TRAF6, PLAUR, CIITA, THBD, PROM1, MT-TL1, IFNB1, SLC31A2, THBS1, HSPD1, TXN, F5, GSR, ANPEP, ITGB4, KCNN4, CTSD, ELANE, ABCG2, HNF4A, TJP1, FGF1, LDHA, IDH1, EIF2AK2, ALOX5, PML, ABCC1, VDR, LDLR, TYMP, SLC11A2, ESR2, SERPINA1, PGR, TYMS, MBP, MIR210, AHR, RANBP2, CEBPB, TIMP1, PRKCB, NTRK2, AKAP9, FLNA, PKM, GADD45A, MT-CO1, VCP, PTPN6, ATF4, COL1A1, HSPA8, XBP1, HSF1, KRT14, IL1RN, SELL, PRL, SOCS3, TGFBI, MMP14, CTSB, SCN4A, CYP3A4, MAP3K5, MAPK9, XRCC1, ACTB, IDO1, CACNA1A, XPO1, BECN1, TOP1, CCL4, RUNX2, KRT8, PLA2G6, CCN2, DNM1L, CD47, FUS, PPARGC1A, S100B, CD209, TFEB, P2RX7, ITGAV, REL, DRD2, CD86, TOP2A, MMP3, APEX1, EZR, KIR3DL1, CYP2A6, IRF8, KCNJ2, BGLAP, TFAP2A, SFPQ, FCGR2A, SLC6A3, PPARA, PGK1, LEPR, ADIPOQ, LGALS1, AOC2, TLR7, MIR98, CD38, KRT7, MTHFR, KCNJ5, RPS27A, NOS1, SERPINA3, CD70, ZBTB16, CYP1B1, HTT, GNAS, KRT19, NRP1, TARDBP, SYP, GPC3, MC1R, MIR130A, TTR, ARNT, GPT, PINK1, MALAT1, GLI1, ADCYAP1, PIK3CB, CDC6, SNAI1, MS4A1, APOB, NME1, DIABLO, KNG1, ADAM17, ACTC1, RACK1, PXN, LPL, BMI1, HBEGF, C3, COX5A, PLG, XRCC5, TNFRSF11B, PDPK1, FLNC, DNAH8, ATF2, ADAM10, MAP2, PSMB4, PRKACA, TWIST1, GSTM1, SPARC, PTPN1, POMC, MT3, BNIP3, PAM, OPTN, GP1BA, GSN, APOA1, SDHA, ALPP, VTN, IL1R1, IDH2, HBG2, XRCC6, CA9, CFH, GLUL, PLAT, PNP, RHD, LBR, AGER, BBC3, PLA2G2A, ODC1, ROCK1, SCO1, WRN, CASK, GRP, F2R, PTGS1, RARB, AGT, ACP1, CYP2D6, MT-CYB, PRKCH, CYP2E1, TG, ENO1, RECK, SDHD, TIMP2, TOP2B, AGTR1, CHAT, ANG, HYOU1, AP1S1, PRTN3, OLR1, EIF4E, PON1, CYP1A2, ABCC2, TLR9, SERPINF1, MCAM, SNTA1, XDH, UGT1A1, F2RL1, CYP19A1, COL2A1, MYL2, ABCB6, RXRA, SREBF1, CYP2C19, RELB, CDC7, NTRK3, NEDD4, MAOA, TKT, GRIN1, SGK1, NR1H4, SLC33A1, GRIN2B, CCK, PTGER2, CCR1, TUBB, HSP90B1, ACE2, MAP2K2, GCG, MAP2K4, CXCR2, MAP3K14, MIR122, KCNE2, TYRP1, RPSA, NR1H2, AP1B1, IGF2R, DHFR, ANXA1, ACHE, IAPP, EPHB2, CRYAB, SCN8A, MMP7, TECRL, BACE1, NT5E, NPPA, S100A8, S100A4, C9orf72, SERPINC1, HMGCR, SCN4B, TIMP3, CXCL9, PTGER4, HBA1, HP, NEU1, DUSP1, ELAVL1, SLC2A4, EGLN2, CDC45, ALPL, PLA2G4A, NEAT1, PRDX1, HOTAIR, LYST, SPHK1, ABCB4, SSTR2, SMN1, NDRG1, ITPR3, RFX5, MFN2, SLC6A4, OSM, MAX, TBX5, PSEN2, MEG3, SDC1, CNTF, BCS1L, TAC1, NTF3, HPRT1, SLC25A4, IGFBP2, NPY, MB, HAMP, ACP5, CCR3, NEDD4L, AQP1, LTF, ADCY10, KIR2DL1, YWHAQ, MSR1, CXCL1, REN, GPX1, CD68, TRPM2, TRMU, KLK3, VIP, EIF2S1, MT2A, G6PC1, CUL3, ABCA1, NAMPT, COMT, PDGFA, LRP1, FASN, DTNBP1, DNASE1, MDK, NSD1, OXT, CHRNA7, CX3CL1, SLC17A5, PTX3, PLA2G7, PIK3C2A, KRT10, SLC40A1, APBB1, LAMP2, ELK1, RHO, PHLDA1, COL8A2, TACR1, OPA1, ACTG1, IGFBP1, ATXN2, C4B, FURIN, CGA, UCP2, GNRH1, CTSK, CRYAA, GAST, HIF1AN, ALDH1A1, SCNN1A, STMN1, ECE1, C1QBP, FGA, GHRL, MT-CO2, SLC3A2, SLC8A1, PRODH, DCN, CXCR1, ATXN3, NAGLU, F8, SOD3, IGHG1, MVP, HADHB, RBX1, MADCAM1, SETX, PTPN3, P4HB, CFL1, PTPA, SCGB1A1, AHCY, COL4A1, GREM1, CSTB, KRIT1, LOXL2, TFF1, MT-ATP6, HNRNPH1, CBS, KRT1, IVL, PPIA, GGT1, H4C16, FTH1, CCDC115, ATP6AP1, STEAP3, GPI, GRHL2, DCT, ALDH2, GAS5, CYP2C9, PGM3, CYBB, HK2, ATP8B1, CALB2, GSTT1, TRPV1, CAPN1, SFTPB, CTSL, KIF1B, CMA1, RAB7A, PIN1, ELOC, LMNB1, RPS6KA1, ADM, PRKAA2, DDC, RETN, ALOX12, GPX4, AVP, RRAS, LITAF, DAG1, SLC1A2, NPHS1, CUL1, ITGA1, STXBP1, SLC4A11, MTHFD1, S100A1, DCTN1, MT-CO3, GUSB, HSP90AB1, NAT2, PAK4, PRDX2, CLEC7A, PEX6, HTR3A, CA2, CHI3L1, NOG, CANX, CST3, SIK2, HELLS, GPHN, PLEK, CYP2B6, ABCC6, PTH, HPS6, TMPRSS6, BAG3, LAMP1, TPO, MAP2K7, ATF6, S100A12, SLC1A3, GARS1, FECH, SCARB2, ADAMTS13, ACTN1, FABP4, CUL2, HEPH, SORL1, GRIN2A, KIR2DS4, GLUD1, AP5M1, NR1I2, EEF1A1, PPBP, ADAM9, ATP6V0A2, GAL, ADPRS, PPP1R15A, COL7A1, CBFB, APOH, SCT, ALAD, KCNJ10, SNX27, BTRC, CSTA, NEFL, CEL, CHMP2B, SCARB1, GLRX, PMM2, PROCR, S100A6, IDUA, PRKCG, RBP4, MTR, PEBP1, YWHAB, OAS1, GCLC, RPE65, CEP290, KRT13, COPA, SKP1, IMMT, TRAF4, CFI, SLC7A11, CTSG, PSTPIP1, ATP6AP2, CLDN4, GAD1, CSNK2B, IBSP, ARG1, MTF1, PDK1, NQO2, PVALB, FKBP1A, S100A13, SURF1, HRH1, PROC, PRDX5, SGCB, FGR, HPS5, CARD16, MT1A, HNRNPA2B1, FOLH1, CPOX, CNP, MIR375, SYNE1, LTBP1, DNTT, POR, PRDX6, TFF3, ALPG, GAA, C5, EPHX1, NAIP, VIPR1, IGLL1, TK1, ADAMTSL1, GJB1, GAP43, COL1A2, PPARD, BLZF1, UGT1A6, KAT2B, F10, GLO1, SLC22A5, LYVE1, TFAM, MAOB, TSPO, CYB5R3, MSX2, FCGR1A, CYBA, SFTPD, CSPG4, AOC4P, MDH2, NUP98, CACNA1S, AMACR, EPS15, TUBB4A, ABCC4, SLPI, LAMB1, ABCC3, AQP3, CTRL, SELENON, AKR1B1, FGF23, CYP3A5, TPT1, RORC, MIR139, COX4I1, MIRLET7A1, PLA2G1B, MSMB, AIF1, MEMO1, PPP3CA, LCAT, SFTPA1, MIR30B, ABCG1, LIPC, EIF5A, LY96, SEC23B, SOAT1, LRP2, DFFB, MVK, CCL26, PIK3C3, IGFBP4, SATB1, HTRA1, NFKBIB, TMPO, RAB5A, ERAS, APOD, INPPL1, GRPR, OVOL2, PNOC, IRF2, PTBP1, UBE2L3, CGB5, SLC39A8, PTGER3, TMPRSS2, GCH1, SLC30A10, VPS33B, MCM5, GLS, FXN, PAH, STIP1, CYP27B1, MTTP, GCGR, MIR125B1, COX19, CARD9, HPGD, UBE2N, LPA, PAFAH1B1, MGP, GPR65, EFEMP1, EHMT2, HPS3, AKR1A1, LIN28B, NTF4, CCDC22, COG2, AQP2, CITED2, HARS1, NDP, MBD4, SELPLG, PRDX3, PPIG, NEXN, COL4A3, SESN2, GNAI1, FTL, XK, IREB2, GLMN, SLC25A20, LOXL1, TFDP1, POGZ, POU2F1, CETP, OTC, CLDN5, RNF7, MIR133A1, SRSF1, S100A7, CUL4A, SATB2, COQ2, SNCAIP, PDIA3, ANXA4, CD160, MMACHC, F9, GSS, MMP8, CALCR, HRG, SERPING1, HINT1, ULK1, ESD, SCD, AVEN, TFR2, GPC1, COL8A1, BNIP3L, HOXA10, SRPK1, FOXO4, POLB, GSTM3, SLC12A2, IGFBP7, HSPB2, SPG7, GLRA1, FDPS, ACP3, PGM1, PON2, ALAS2, FLOT2, S100A2, PNPLA6, AFG3L2, PSENEN, GOLM1, PIP5K1A, M6PR, APOC3, ABCB7, RXRB, PSMB5, CHKA, OXA1L, DUX4, CACNA1H, COG6, MAP1LC3A, ARF1, CLDN3, CYSLTR1, ACO1, CDH11, CLEC4M, ZMPSTE24, FBLN5, MT-RNR1, SLC4A4, SEPTIN7, FAM107A, NNMT, NR5A2, PPIF, SHBG, COL5A1, CBX5, ALG2, INTS3, IGF2BP2, SLC30A8, DHPS, B4GALT1, TRPC6, HPR, CUL4B, EIF6, KRT2, CLSPN, CTBP1, LRRC8A, CLIC1, GMNN, IGF2BP3, OCA2, FKBP4, APLP2, PDLIM5, IDE, ADH1C, TRIM22, LOXL4, CRABP2, INTS2, ADK, HPX, FLOT1, MYO5B, ERF, SLC22A2, HRH2, TMSB4X, SREBF2, PCSK2, H1-4, LNPEP, SYNGAP1, KAT2A, BLOC1S1, MT1X, DROSHA, EIF4A1, CCL27, HNMT, HDC, ESRRA, DUSP4, MAPKAPK5, STS, BCHE, PLSCR1, HNRNPL, P2RX4, PTPRU, PROS1, PTAFR, H3C14, FGF13, VPS35L, SERPINF2, CPQ, MPP1, PSMD6, SNCG, GPX3, CIZ1, ZNF687, LALBA, MSX1, CTSA, VPS35, SLC26A4, PRDM2, CDT1, GJB3, UGT1A9, PRND, CSN1S1, HSD17B10, PON3, SUOX, NOC2L, ZNF469, SMURF1, PNKP, ABCD1, CAVIN1, NUDT1, ATP2B4, SLC25A3, UQCRFS1, AQP5, NRF1, APLP1, PARVA, HPS1, NISCH, SETDB1, PANK2, FARSB, SLCO1B1, SYT1, SLC5A1, MAP1LC3B, DPAGT1, PER1, RBM8A, LPXN, ST3GAL4, SLC6A2, CUL5, PYY, A2M, AP4M1, WASL, SLC25A1, SLC35C1, PSME1, HDGF, GNE, GBF1, ETFDH, GAS1, GRIN2D, CS, TXN2, SLC11A1, AHSG, INTS4, AKR1C1, SERPINB1, MIA2, HJV, NPHS2, SLC26A2, C1S, SLC39A14, PCBP1, GPR143, EFEMP2, STEAP4, APOA4, MOXD1, SUMF1, LBP, LDHB, TALDO1, DCTN4, CLCN3, CTNS, MT1G, UGT1A4, CTSE, RPS2, LYAR, TULP1, ALS2, PGAM1, MT1E, APOA2, ALDH9A1, MELTF, TFPI, PYCR1, UGT1A, VPS13A, CA4, SORD, SLC46A1, PPP1R8, HRK, GBE1, PLA2G10, DNM1, FOXF1, CUX2, SNAP29, HPS4, AHSP, KRT3, COA6, DYRK1B, RANBP1, FKBP6, XAF1, HSPA14, PGD, FAAH, STEAP2, AZI2, GRIN2C, CFB, F12, CCL16, SLC25A6, ADNP, SEPSECS, VSX1, ANXA6, DOLK, APOC2, MPLKIP, BLOC1S3, LYZ, TDP2, OGDH, ADPRH, CA12, H6PD, SPATA5, CCDC93, LIPG, ATP13A2, SCN3A, ITGB1BP1, LIPE, ALG13, SOST, HMOX2, ADAM8, ATP5F1D, SCG5, CYB5A, PPP5C, DSPP, MOGS, ADH1B, ILF2, SLC39A4, VSX2, MT1F, FMO3, TIE1, SELENOP, UBE2C, COL4A4, CCBE1, COX15, LOXL3, LGALS3BP, LTBP3, GRIA3, TIMP4, CAMK1D, UGDH, CANT1, DAO, SLC51B, UBIAD1, KRT12, NCAPH2, SELENBP1, SLC30A1, DPM1, CPE, NAGA, TAC3, DCAF8, ALG1, KCNN2, MPI, HULC, MDH1, TAGLN2, ADRB3, MBD2, SPTBN2, COX10, TONSL, PTRH2, TP73-AS1, PAEP, CSMD1, AMT, TFPT, BLVRB, SERPINA6, GPX7, ALDH7A1, SKAP2, MT4, ALG12, KIF7, S100A5, UGT1A3, LEPQTL1, LACTB, PTGES3, MAN2A1, AKR1C2, SLC30A2, COASY, STT3B, CUL7, SLC1A4, GRIN3B, TRIM63, ASIP, PHKG1, GC, VRK2, CLEC4E, MIR26B, ACY1, PTS, ATP5F1B, SPATA2, ATP6V1A, FAH, PENK, RNASE1, ATP6V0A1, C9, FLNC-AS1, INTS5, GOT1, USP22, ACTN3, IGKC, NALCN, NMNAT1, HNRNPH3, GART, KIF1C, TMSB10, KRT9, MRPL41, UTS2, SPART, SI, MAT2A, CBLIF, SIGLEC10, ADRA1A, NPR1, PLEKHA7, TRPA1, SF3A2, ATG9A, BMP1, FABP5, STT3A, SLC26A5, NTSR1, CES1, CYP51A1, COQ8A, COQ4, KLC2, PLOD1, UFD1, GABPA, SLC22A1, HLTF, H3C15, DDOST, EYS, MCM8, SUCLG1, PDP1, KCNJ12, LTBP4, PLTP, GRM3, KCNJ3, ECHS1, CEP104, RAB3GAP1, SLC30A5, CAND1, SLC16A3, LRPAP1, CLEC4D, ALDH18A1, CYP2C8, C1QA, MBTPS1, ASGR2, C19orf12, COX16, SHMT1, AGFG1, FABP1, SRD5A3, PI4KB, RNF4, ULK2, GABBR1, SHMT2, COX4I2, COX6B1, TGOLN2, SULT1A3, ATP6V1D, MIP, PTGIS, GNRH2, GLDC, AP1S2, EDN2, CSRP1, MIR495, AOX1, UBE2V1, RPLP2, CRMP1, SRSF6, GPR18, NMT1, COG7, SLC26A1, REG1A, SCARF1, GPAM, WHRN, GATA5, NGB, PSG2, MPDU1, MT1H, SMOX, GPLD1, SLC6A1, MMP16, ALG9, PTGER1, TMEM165, WDR45, FBLN2, CTH, SLC2A10, ADH5, CYB561, WASHC1, INTS6, NEIL2, SULT1A1, C8B, UGT2B7, VKORC1, DLD, APCS, AZGP1, RGN, RXRG, ALG6, PNPLA3, PCSK7, LINC01194, GOT2, SERPIND1, ORC4, KRR1, SUCLA2, PPID, LTA4H, GPX2, CSN2, CYP2A13, NORAD, CPSF3, RPL21, PPP4C, TIMM8A, MGME1, KHDRBS3, PYM1, GLRB, KDM4A, FDFT1, STEAP1, SUMO2, ARFRP1, NCBP2, MAT1A, COG4, COPZ1, SLC39A1, LAP3, RNASET2, COL10A1, TXNDC5, AKAP1, GOSR1, SCAMP5, GLRX3, KCNJ18, DPM2, SLCO1B3, FABP2, MID1, PRSS1, ASPA, NEIL1, TNXA, ADI1, MPP2, CYC1, INTS7, MLC1, POMP, LACC1, APPBP2, PITRM1, LCT, PPOX, ASIC1, OAS2, KCNK9, SIGLEC9, GJB4, GRINA, FLRT2, IFNL3, EHD2, ANXA3, CLEC3B, ATL1, MSRA, VDAC2, APBA1, GTF2B, CLPB, ATP2C1, MBD6, LRG1, COX7B, SCP2, H3C13, SNCB, KIFC1, DLAT, SLC15A1, ISCU, COX6A1, MLN, GTF3A, ARL2, HTN3, RSU1, ALPI, PIGF, CLEC6A, MFAP2, PYCR2, TUBA3C, SLC10A2, STAB1, MT1M, AS3MT, TGM3, H2BC12L, SULT1E1, IDH3A, ACAT1, GRIN3A, LUM, OAZ1, MREG, KCNV2, ITGBL1, ERFE, ORC6, PCDH12, MTREX, SCNN1B, DIO3, ALDH3A2, XRN2, MTHFD1L, SLC24A3, C4BPA, COG5, MED25, MYT1, ERVK-6, GTPBP2, ATP6V1E1, CTSH, SLC35A2, FTSJ1, COX8A, PHPT1, COG1, SSR4, NFE2L1, SOAT2, ALG3, COX7C, GGCX, COX6C, CUTA, TMC1, SLC35A1, MGAM, SLC25A37, AP1M2, RNF19A, SC5D, MT1B, COMMD2, AGXT, CLCN4, ARSH, NDUFA4, CHST6, P2RX2, CA1, MBD1, GORAB, AASS, SLC6A9, DUSP3, CA3, CYP4B1, ACAA2, SCNN1G, FTMT, UQCC2, IDI1, LYRM4, CYBRD1, LPO, COMMD3, REXO2, S100A14, BHMT, APEH, RTF1, SLC38A4, NTAN1, ELAVL2, CYP2J2, MTHFD2, KCNJ4, KLF8, SOSTDC1, RHCG, B4GALT5, KLK7, BTAF1, SLC45A2, GNAZ, SLC6A5, TMOD1, RPRM, DHDH, NDUFB8, HCAR1, CRIP2, ALG11, MEOX2, MAN1C1, SEC61B, TMPRSS11A, SLCO1A2, C1QC, NPLOC4, AUH, AGL, SLC22A6, IFNA8, SNAP91, TOP1MT, COMMD4, COPG1, SLC5A8, PUM3, GATB, NAPRT, RAB9A, KLKB1, DPM3, IGFALS, ITPRID2, BAZ2B, AANAT, NFU1, ALAS1, SNX14, CPD, RTN1, GREM2, CYP2S1, MAN1B1, GSTO2, ACYP2, RNF14, COG3, GUK1, SERPINA7, HCAR2, MOCS2, GATM, SQLE, HCCS, H2BC4, CYP3A7, KCTD1, SRM, GFM2, RAB18, AGRP, PORCN, RNPEP, SLC35A3, SOX15, PDZD11, GSTK1, BRAP, SMS, GOLGB1, FA2H, TREH, PTDSS1, COMMD10, ELAVL3, DGAT2, STX6, APEX2, NFS1, PLEKHA5, DHRS7B, DPP3, GMPPA, IGHG4, SHANK3, PEDS1, ACR, DUX4L1, COX6A2, RIN2, DZANK1, GNMT, PGAM4, ATP6V0C, CHCHD4, S100G, APBA2, COX7A1, EPHA6, UCN3, ALDH1A3, SEPHS1, REPS1, CLPP, INTS8, TIMM23, HACL1, COG8, SLC36A1, HSPA13, GET3, SLC32A1, INTS10, TIMM13, COA3, SULT2A1, POLR3F, SLC9A8, HAGH, SARDH, KYNU, SLC39A5, MT1DP, CNNM4, RPH3A, LRRC47, NDUFA5, SUMF2, UCK2, TXNL1, INPP5J, C1QB, GRHL1, COX20, NCDN, MYG1, ZACN, SPATA22, SRXN1, SLC28A1, MSRB2, SLC30A6, ALDH1B1, METTL17, RHBG, PET117, HAAO, RNLS, PLEKHA6, MTCO2P12, GCAT, RBM4B, GTF2A1, HCAR3, COX7A2, MORF4L2, PANK1, UQCRC1, ALG14, FNTB, IL22RA2, NAA25, GLRA2, NKX2-3, QPRT, PLA2G2E, SNAI3, LCN1, RASL12, SLC22A8, STXBP4, TPPP3, DMGDH, GPRC6A, ACAD10, OMP, SLC35A5, MSRB1, UGT2B17, SERPINA4, HAO1, BIRC8, SHOX2, ITIH2, GALNT13, SLAIN1, DCAF17, GMFB, OPTC, GSTA4, AGMAT, DAND5, VPS29, CFAP45, BAAT, MEIOB, PM20D2, SUCLG2, SLC5A4, CNNM3, CNNM2, CRIP1, GLYAT, KRT82, CRYGD, LOC108663987, AFM, CYP2F1, CYP2C18, KCNJ14, GLRA3, KYAT1, NEK5, CROT, ZNF133, HSP90AA2P, SERPINA12, BHMT2, PAOX, SLC6A12, A1BG, GCSH, CYP4F2, COA7, PTMS, HNF4G, SLC30A4, PDF, PIPOX, CNNM1, TRERNA1, AQP10, AMY2B, ACSM3, PYROXD1, PCTP, GALNT4, CYP4Z1, ZNF711, COA4, MMGT1, SYNGAP1-AS1, DMAC2L, SIMC1, HTN1, IDNK, CMC2, AGXT2, SLC35A4, PAFAH2, TMEM177, ELOF1, OR2AG1, MIR5688, CA7, ATHS |
| --- |
